# Supplementary material for: Hot spot prediction in protein-protein interactions by an ensemble system
Source: BMC Syst Biol. 2018 Dec 31;12(Suppl 9):132. doi: 10.1186/s12918-018-0665-8 (PMC6311905; doi:10.1186/s12918-018-0665-8)
Supplement: Supplementary file 2 — All relevant MATLAB code. A simple Matlab implement of our predictor. (ZIP 19,742 kb) [file 12918_2018_665_MOESM2_ESM.zip › Additional file 2/Read ASA/ASA website/dbMPIKT/2X0BB.html]

 NetSurfP - Protein Surface Accessibility and Secondary Structure Predictions

  

|  |  |  |
| --- | --- | --- |
|  |  | NetSurfP - Protein Surface Accessibility and Secondary Structure Predictions  Technical University of Denmark |

  


---

```
# For publication of results, please cite:
# A generic method for assignment of reliability scores applied to solvent accessibility predictions.
# Bent Petersen, Thomas Nordahl Petersen, Pernille Andersen, Morten Nielsen and Claus Lundegaard
# BMC Structural Biology 2009, 9:51 doi:10.1186/1472-6807-9-51
#
# Column 1: Class assignment - B for buried or E for Exposed - Threshold: 25% exposure, but not based on RSA
# Column 2: Amino acid
# Column 3: Sequence name
# Column 4: Amino acid number
# Column 5: Relative Surface Accessibility - RSA
# Column 6: Absolute Surface Accessibility
# Column 7: Z-fit score for RSA prediction
# Column 8: Probability for Alpha-Helix
# Column 9: Probability for Beta-strand
# Column 10: Probability for Coil
E D  2X0BB                  1    0.833 120.021  -0.077   0.003   0.003   0.994
E R  2X0BB                  2    0.335  76.738   1.019   0.021   0.451   0.528
B V  2X0BB                  3    0.127  19.551  -1.427   0.003   0.718   0.279
B Y  2X0BB                  4    0.105  22.460   0.374   0.018   0.846   0.136
B I  2X0BB                  5    0.093  17.113  -0.761   0.018   0.846   0.136
B H  2X0BB                  6    0.114  20.700   1.333   0.022   0.552   0.426
B P  2X0BB                  7    0.119  16.829  -0.272   0.022   0.359   0.619
B F  2X0BB                  8    0.085  17.019  -0.319   0.022   0.359   0.619
B H  2X0BB                  9    0.119  21.573  -0.196   0.074   0.484   0.442
B L  2X0BB                 10    0.052   9.558   0.261   0.079   0.592   0.329
B V  2X0BB                 11    0.097  14.878   0.152   0.087   0.683   0.230
B I  2X0BB                 12    0.097  17.945  -0.189   0.087   0.683   0.230
B H  2X0BB                 13    0.202  36.817  -0.164   0.021   0.451   0.528
E N  2X0BB                 14    0.435  63.713  -0.579   0.021   0.279   0.699
E E  2X0BB                 15    0.557  97.273  -0.475   0.020   0.205   0.775
E S  2X0BB                 16    0.450  52.787  -0.794   0.064   0.216   0.721
E T  2X0BB                 17    0.490  67.908   0.173   0.064   0.216   0.721
B C  2X0BB                 18    0.194  27.195  -0.702   0.125   0.227   0.648
E E  2X0BB                 19    0.480  83.891  -0.112   0.118   0.150   0.732
E Q  2X0BB                 20    0.564 100.802   0.424   0.199   0.152   0.649
E L  2X0BB                 21    0.264  48.357   0.015   0.307   0.165   0.527
E A  2X0BB                 22    0.476  52.510  -0.650   0.278   0.093   0.628
E K  2X0BB                 23    0.566 116.323   0.119   0.191   0.086   0.723
E A  2X0BB                 24    0.542  59.739  -0.232   0.191   0.086   0.723
E N  2X0BB                 25    0.559  81.764  -0.500   0.191   0.086   0.723
E A  2X0BB                 26    0.557  61.414  -0.796   0.113   0.087   0.800
E G  2X0BB                 27    0.631  49.636  -0.631   0.053   0.043   0.903
E K  2X0BB                 28    0.493 101.472   0.831   0.052   0.084   0.864
E P  2X0BB                 29    0.357  50.687  -0.796   0.056   0.142   0.802
E K  2X0BB                 30    0.493 101.348  -0.657   0.056   0.142   0.802
E D  2X0BB                 31    0.634  91.359  -0.697   0.052   0.084   0.864
E P  2X0BB                 32    0.322  45.763  -0.358   0.052   0.084   0.864
E T  2X0BB                 33    0.502  69.669  -1.227   0.019   0.141   0.840
E F  2X0BB                 34    0.286  57.340  -0.099   0.019   0.141   0.840
E I  2X0BB                 35    0.481  89.022  -0.259   0.018   0.088   0.893
B P  2X0BB                 36    0.280  39.732  -1.940   0.018   0.088   0.893
E A  2X0BB                 37    0.350  38.570  -0.946   0.018   0.088   0.893
E P  2X0BB                 38    0.337  47.834  -1.591   0.018   0.088   0.893
B I  2X0BB                 39    0.198  36.593  -1.215   0.018   0.047   0.935
E Q  2X0BB                 40    0.513  91.568  -1.571   0.018   0.047   0.935
E A  2X0BB                 41    0.553  60.930  -1.244   0.018   0.019   0.964
E K  2X0BB                 42    0.675 138.868  -1.446   0.018   0.019   0.964
E T  2X0BB                 43    0.444  61.541  -1.840   0.018   0.019   0.964
E S  2X0BB                 44    0.440  51.533  -1.075   0.005   0.015   0.979
E P  2X0BB                 45    0.397  56.320  -0.445   0.018   0.019   0.964
E V  2X0BB                 46    0.598  91.897  -0.970   0.018   0.019   0.964
E N  2X0BB                 47    0.713 104.456  -0.664   0.058   0.017   0.925
E E  2X0BB                 48    0.539  94.111  -1.173   0.058   0.017   0.925
E K  2X0BB                 49    0.650 133.808  -0.484   0.058   0.017   0.925
E A  2X0BB                 50    0.570  62.858  -0.672   0.058   0.017   0.925
E L  2X0BB                 51    0.291  53.209  -0.504   0.053   0.043   0.903
E Q  2X0BB                 52    0.550  98.159  -0.228   0.053   0.043   0.903
E D  2X0BB                 53    0.662  95.437  -0.379   0.053   0.043   0.903
E Q  2X0BB                 54    0.650 116.036  -0.800   0.053   0.043   0.903
E L  2X0BB                 55    0.566 103.580  -0.883   0.053   0.043   0.903
E V  2X0BB                 56    0.375  57.591  -0.447   0.113   0.043   0.844
E L  2X0BB                 57    0.490  89.811  -0.849   0.113   0.087   0.800
E V  2X0BB                 58    0.487  74.883  -1.049   0.118   0.150   0.732
E A  2X0BB                 59    0.327  36.068  -1.220   0.118   0.150   0.732
E A  2X0BB                 60    0.531  58.516  -0.518   0.118   0.150   0.732
E K  2X0BB                 61    0.615 126.485  -0.116   0.052   0.084   0.864
E L  2X0BB                 62    0.368  67.436  -0.225   0.053   0.043   0.903
E D  2X0BB                 63    0.484  69.759  -0.081   0.053   0.043   0.903
E T  2X0BB                 64    0.696  96.577  -0.563   0.181   0.016   0.803
E E  2X0BB                 65    0.713 124.491  -0.621   0.181   0.016   0.803
E D  2X0BB                 66    0.483  69.644  -1.006   0.246   0.004   0.750
E K  2X0BB                 67    0.493 101.348   0.256   0.430   0.016   0.555
E L  2X0BB                 68    0.484  88.694  -0.130   0.694   0.003   0.303
E R  2X0BB                 69    0.318  72.845   0.368   0.782   0.003   0.216
B A  2X0BB                 70    0.144  15.847  -0.606   0.923   0.002   0.076
E A  2X0BB                 71    0.447  49.281  -0.139   0.923   0.002   0.076
E M  2X0BB                 72    0.399  79.760   1.282   0.923   0.002   0.076
B V  2X0BB                 73    0.043   6.671   0.371   0.970   0.001   0.030
B G  2X0BB                 74    0.164  12.875   0.054   0.923   0.002   0.076
E M  2X0BB                 75    0.477  95.528   0.674   0.970   0.001   0.030
B L  2X0BB                 76    0.106  19.372  -0.072   0.970   0.001   0.030
B A  2X0BB                 77    0.083   9.147  -0.668   0.970   0.001   0.030
B N  2X0BB                 78    0.147  21.492  -0.099   0.970   0.001   0.030
E F  2X0BB                 79    0.437  87.706   1.413   0.970   0.001   0.030
B L  2X0BB                 80    0.059  10.858   0.775   0.970   0.001   0.030
B G  2X0BB                 81    0.018   1.385   0.448   0.970   0.001   0.030
B F  2X0BB                 82    0.149  29.844   0.080   0.970   0.001   0.030
E R  2X0BB                 83    0.397  90.821   1.539   0.988   0.000   0.012
B I  2X0BB                 84    0.041   7.585   0.348   0.988   0.000   0.012
B Y  2X0BB                 85    0.065  13.869   0.097   0.970   0.001   0.030
E G  2X0BB                 86    0.426  33.534   1.757   0.970   0.001   0.030
E M  2X0BB                 87    0.301  60.170   1.262   0.970   0.001   0.030
B H  2X0BB                 88    0.032   5.894   0.460   0.970   0.001   0.030
E S  2X0BB                 89    0.281  32.875   0.533   0.923   0.002   0.076
E E  2X0BB                 90    0.653 114.044   0.495   0.858   0.002   0.139
E L  2X0BB                 91    0.278  50.865  -0.596   0.694   0.003   0.303
E W  2X0BB                 92    0.393  94.420  -1.001   0.406   0.004   0.590
E G  2X0BB                 93    0.689  54.264  -0.479   0.176   0.004   0.820
E V  2X0BB                 94    0.488  74.990  -0.643   0.053   0.005   0.942
E V  2X0BB                 95    0.519  79.693  -0.958   0.016   0.005   0.979
E H  2X0BB                 96    0.556 101.209  -0.820   0.003   0.003   0.994
E G  2X0BB                 97    0.338  26.593   1.427   0.005   0.015   0.979
B A  2X0BB                 98    0.037   4.055  -0.437   0.021   0.279   0.699
B T  2X0BB                 99    0.029   4.036   1.008   0.001   0.900   0.099
B V  2X0BB                100    0.024   3.704   0.336   0.001   0.959   0.040
B L  2X0BB                101    0.026   4.761   0.872   0.001   0.900   0.099
B S  2X0BB                102    0.028   3.317   0.575   0.022   0.359   0.619
B P  2X0BB                103    0.057   8.074  -0.860   0.717   0.014   0.269
B T  2X0BB                104    0.150  20.819  -0.335   0.858   0.002   0.139
B A  2X0BB                105    0.056   6.193  -0.250   0.879   0.010   0.111
B V  2X0BB                106    0.029   4.473  -0.014   0.975   0.003   0.022
B F  2X0BB                107    0.153  30.667  -0.185   0.970   0.001   0.030
B G  2X0BB                108    0.109   8.539   0.113   0.970   0.001   0.030
B T  2X0BB                109    0.034   4.702  -0.758   0.970   0.001   0.030
B L  2X0BB                110    0.050   9.155   0.010   0.988   0.000   0.012
B A  2X0BB                111    0.099  10.921  -0.739   0.988   0.000   0.012
B S  2X0BB                112    0.066   7.723   0.096   0.970   0.001   0.030
B L  2X0BB                113    0.031   5.731   0.327   0.970   0.001   0.030
B Y  2X0BB                114    0.141  30.174   0.247   0.938   0.007   0.055
B L  2X0BB                115    0.210  38.451  -0.402   0.802   0.014   0.185
B G  2X0BB                116    0.119   9.381  -2.394   0.502   0.002   0.495
B A  2X0BB                117    0.072   7.989  -1.885   0.109   0.005   0.886
E L  2X0BB                118    0.516  94.480   0.699   0.016   0.005   0.979
E D  2X0BB                119    0.545  78.578  -1.201   0.246   0.004   0.750
E H  2X0BB                120    0.601 109.413   0.556   0.622   0.015   0.363
B T  2X0BB                121    0.084  11.692  -0.481   0.879   0.010   0.111
B A  2X0BB                122    0.224  24.674   0.770   0.970   0.001   0.030
E D  2X0BB                123    0.565  81.445   1.435   0.970   0.001   0.030
B R  2X0BB                124    0.191  43.739   1.220   0.988   0.000   0.012
B L  2X0BB                125    0.031   5.768  -0.057   0.988   0.000   0.012
E Q  2X0BB                126    0.306  54.634   0.764   0.970   0.001   0.030
E A  2X0BB                127    0.563  61.999   0.842   0.970   0.001   0.030
B I  2X0BB                128    0.113  20.923  -0.726   0.923   0.002   0.076
B L  2X0BB                129    0.123  22.595  -0.566   0.694   0.003   0.303
E G  2X0BB                130    0.571  44.930  -0.269   0.176   0.004   0.820
B V  2X0BB                131    0.172  26.436   0.089   0.058   0.017   0.925
E P  2X0BB                132    0.501  71.149  -0.369   0.018   0.047   0.935
E W  2X0BB                133    0.445 106.926  -1.369   0.053   0.043   0.903
E K  2X0BB                134    0.627 128.912  -1.206   0.113   0.043   0.844
E D  2X0BB                135    0.539  77.684  -1.215   0.115   0.016   0.868
E K  2X0BB                136    0.499 102.603  -0.769   0.181   0.016   0.803
E N  2X0BB                137    0.582  85.161  -1.132   0.184   0.043   0.773
E C  2X0BB                138    0.358  50.221  -0.696   0.268   0.043   0.689
E T  2X0BB                139    0.459  63.649  -1.152   0.268   0.043   0.689
E S  2X0BB                140    0.412  48.298  -1.226   0.354   0.048   0.598
E R  2X0BB                141    0.396  90.798  -1.003   0.455   0.046   0.498
E L  2X0BB                142    0.435  79.703  -1.571   0.522   0.016   0.462
E D  2X0BB                143    0.492  70.926  -0.776   0.622   0.015   0.363
B A  2X0BB                144    0.202  22.260  -1.306   0.717   0.014   0.269
E H  2X0BB                145    0.433  78.817  -0.850   0.717   0.014   0.269
E K  2X0BB                146    0.481  98.839   0.074   0.717   0.014   0.269
B V  2X0BB                147    0.134  20.534  -0.840   0.879   0.010   0.111
E L  2X0BB                148    0.318  58.299  -0.305   0.858   0.002   0.139
E S  2X0BB                149    0.533  62.491  -0.030   0.923   0.002   0.076
B A  2X0BB                150    0.149  16.464  -0.248   0.923   0.002   0.076
B L  2X0BB                151    0.142  25.927   0.052   0.923   0.002   0.076
E Q  2X0BB                152    0.571 101.981   0.276   0.970   0.001   0.030
E A  2X0BB                153    0.336  37.027  -0.100   0.923   0.002   0.076
B V  2X0BB                154    0.049   7.562   0.286   0.970   0.001   0.030
E Q  2X0BB                155    0.348  62.206   0.668   0.970   0.001   0.030
E G  2X0BB                156    0.523  41.129   0.815   0.970   0.001   0.030
B L  2X0BB                157    0.089  16.241   0.194   0.970   0.001   0.030
B L  2X0BB                158    0.110  20.196  -0.504   0.970   0.001   0.030
E V  2X0BB                159    0.527  80.923   0.582   0.970   0.001   0.030
E A  2X0BB                160    0.426  46.912   0.500   0.923   0.002   0.076
B Q  2X0BB                161    0.148  26.379  -0.955   0.858   0.002   0.139
E G  2X0BB                162    0.470  37.005  -1.196   0.694   0.003   0.303
E R  2X0BB                163    0.605 138.431  -0.125   0.321   0.003   0.675
E A  2X0BB                164    0.439  48.400  -1.337   0.109   0.005   0.886
E D  2X0BB                165    0.657  94.674  -2.042   0.018   0.019   0.964
E S  2X0BB                166    0.537  62.913  -1.587   0.058   0.017   0.925
E Q  2X0BB                167    0.621 111.000  -0.852   0.113   0.043   0.844
E A  2X0BB                168    0.428  47.166  -0.770   0.268   0.043   0.689
E Q  2X0BB                169    0.532  95.015  -0.346   0.184   0.043   0.773
E L  2X0BB                170    0.304  55.644   0.164   0.216   0.235   0.548
E L  2X0BB                171    0.292  53.520   0.761   0.159   0.516   0.325
B L  2X0BB                172    0.033   6.061   0.484   0.087   0.683   0.230
E S  2X0BB                173    0.351  41.184   1.178   0.085   0.775   0.140
B T  2X0BB                174    0.130  18.045   0.267   0.085   0.775   0.140
B V  2X0BB                175    0.038   5.841  -0.047   0.087   0.683   0.230
B V  2X0BB                176    0.075  11.558  -1.199   0.079   0.592   0.329
B G  2X0BB                177    0.096   7.524   0.535   0.072   0.855   0.073
B V  2X0BB                178    0.020   3.089   0.766   0.085   0.775   0.140
B F  2X0BB                179    0.058  11.721   0.424   0.085   0.775   0.140
B T  2X0BB                180    0.047   6.519  -1.018   0.169   0.612   0.219
E A  2X0BB                181    0.275  30.272  -0.159   0.135   0.317   0.548
E P  2X0BB                182    0.561  79.648  -0.334   0.052   0.084   0.864
E G  2X0BB                183    0.572  44.985  -1.209   0.018   0.047   0.935
B L  2X0BB                184    0.141  25.817   0.205   0.018   0.047   0.935
E H  2X0BB                185    0.552 100.482   0.111   0.018   0.047   0.935
B L  2X0BB                186    0.166  30.413  -1.069   0.053   0.043   0.903
E K  2X0BB                187    0.325  66.935   0.469   0.339   0.016   0.645
E Q  2X0BB                188    0.703 125.574   0.355   0.694   0.003   0.303
E P  2X0BB                189    0.558  79.237   0.968   0.858   0.002   0.139
B F  2X0BB                190    0.057  11.360   0.215   0.923   0.002   0.076
E V  2X0BB                191    0.307  47.124   0.716   0.938   0.007   0.055
E Q  2X0BB                192    0.505  90.264   0.469   0.975   0.003   0.022
E G  2X0BB                193    0.308  24.271   0.210   0.923   0.002   0.076
B L  2X0BB                194    0.051   9.375  -0.263   0.923   0.002   0.076
E A  2X0BB                195    0.334  36.752   0.261   0.879   0.010   0.111
E L  2X0BB                196    0.558 102.243   0.838   0.879   0.010   0.111
B Y  2X0BB                197    0.266  56.887   0.221   0.802   0.014   0.185
B T  2X0BB                198    0.138  19.141  -0.055   0.522   0.016   0.462
E P  2X0BB                199    0.365  51.751  -1.060   0.354   0.048   0.598
E V  2X0BB                200    0.536  82.337  -0.655   0.191   0.086   0.723
E V  2X0BB                201    0.338  51.997  -1.295   0.125   0.227   0.648
E L  2X0BB                202    0.370  67.674  -0.129   0.069   0.386   0.545
B P  2X0BB                203    0.269  38.185  -0.073   0.074   0.484   0.442
E R  2X0BB                204    0.408  93.409  -0.080   0.074   0.484   0.442
B S  2X0BB                205    0.273  31.972  -0.313   0.069   0.386   0.545
B L  2X0BB                206    0.246  45.098  -0.737   0.066   0.296   0.638
E D  2X0BB                207    0.314  45.204  -0.893   0.056   0.142   0.802
B F  2X0BB                208    0.170  34.139  -1.043   0.113   0.043   0.844
E T  2X0BB                209    0.551  76.382  -0.217   0.115   0.016   0.868
E E  2X0BB                210    0.437  76.379   0.177   0.058   0.017   0.925
B L  2X0BB                211    0.218  39.843   0.286   0.782   0.003   0.216
E D  2X0BB                212    0.592  85.379   0.015   0.923   0.002   0.076
E V  2X0BB                213    0.465  71.471   1.505   0.970   0.001   0.030
B A  2X0BB                214    0.023   2.502  -0.190   0.970   0.001   0.030
B A  2X0BB                215    0.141  15.527   0.765   0.988   0.000   0.012
E E  2X0BB                216    0.533  93.185   1.024   0.988   0.000   0.012
B K  2X0BB                217    0.235  48.422   1.188   0.970   0.001   0.030
B I  2X0BB                218    0.015   2.720   1.248   0.970   0.001   0.030
B D  2X0BB                219    0.228  32.812   0.177   0.970   0.001   0.030
E R  2X0BB                220    0.473 108.431   1.056   0.988   0.000   0.012
B F  2X0BB                221    0.111  22.358   0.557   0.988   0.000   0.012
B M  2X0BB                222    0.038   7.704  -0.384   0.988   0.000   0.012
E Q  2X0BB                223    0.399  71.333   0.685   0.970   0.001   0.030
E A  2X0BB                224    0.558  61.481   0.430   0.970   0.001   0.030
E V  2X0BB                225    0.329  50.613   0.466   0.923   0.002   0.076
B T  2X0BB                226    0.145  20.056  -1.604   0.694   0.003   0.303
E G  2X0BB                227    0.662  52.115  -0.013   0.246   0.004   0.750
E W  2X0BB                228    0.599 144.011  -0.960   0.053   0.005   0.942
E K  2X0BB                229    0.485  99.867   0.516   0.053   0.043   0.903
B T  2X0BB                230    0.083  11.498  -0.723   0.113   0.087   0.800
E G  2X0BB                231    0.540  42.459   0.625   0.056   0.142   0.802
E C  2X0BB                232    0.579  81.221  -0.156   0.118   0.150   0.732
B S  2X0BB                233    0.135  15.799  -0.318   0.118   0.150   0.732
B L  2X0BB                234    0.102  18.731  -0.478   0.064   0.216   0.721
E M  2X0BB                235    0.525 104.952  -0.278   0.019   0.141   0.840
E G  2X0BB                236    0.501  39.468  -1.294   0.018   0.088   0.893
B A  2X0BB                237    0.274  30.195  -1.730   0.018   0.047   0.935
E S  2X0BB                238    0.386  45.263  -0.965   0.018   0.019   0.964
E V  2X0BB                239    0.540  83.044  -1.477   0.176   0.004   0.820
E D  2X0BB                240    0.533  76.748  -1.551   0.321   0.003   0.675
B S  2X0BB                241    0.106  12.470  -0.488   0.406   0.004   0.590
B T  2X0BB                242    0.188  26.062  -0.117   0.561   0.047   0.393
B L  2X0BB                243    0.127  23.235   0.770   0.649   0.163   0.188
B A  2X0BB                244    0.077   8.452   0.546   0.594   0.285   0.121
B F  2X0BB                245    0.020   4.074   0.972   0.487   0.370   0.144
B N  2X0BB                246    0.057   8.330  -0.328   0.487   0.370   0.144
B T  2X0BB                247    0.058   8.100  -0.231   0.373   0.401   0.226
B Y  2X0BB                248    0.049  10.407   0.253   0.373   0.401   0.226
B V  2X0BB                249    0.038   5.871  -0.557   0.268   0.505   0.227
B H  2X0BB                250    0.153  27.831   0.670   0.172   0.690   0.138
B F  2X0BB                251    0.062  12.383  -0.338   0.169   0.612   0.219
B Q  2X0BB                252    0.250  44.704   0.056   0.148   0.418   0.435
B G  2X0BB                253    0.122   9.586  -1.180   0.125   0.227   0.648
E K  2X0BB                254    0.393  80.840  -0.286   0.056   0.142   0.802
B M  2X0BB                255    0.069  13.847  -1.083   0.056   0.142   0.802
E K  2X0BB                256    0.471  96.844   0.779   0.056   0.142   0.802
B G  2X0BB                257    0.232  18.290  -1.356   0.018   0.088   0.893
E F  2X0BB                258    0.408  81.866  -0.176   0.018   0.047   0.935
E S  2X0BB                259    0.365  42.813  -1.426   0.053   0.043   0.903
E L  2X0BB                260    0.645 118.136  -0.177   0.052   0.084   0.864
E L  2X0BB                261    0.414  75.748  -0.719   0.052   0.084   0.864
E A  2X0BB                262    0.456  50.273  -0.939   0.052   0.084   0.864
E E  2X0BB                263    0.541  94.548  -0.759   0.064   0.216   0.721
E P  2X0BB                264    0.635  90.135  -0.151   0.066   0.296   0.638
E Q  2X0BB                265    0.346  61.742  -0.169   0.021   0.279   0.699
E E  2X0BB                266    0.533  93.168  -0.122   0.022   0.359   0.619
B F  2X0BB                267    0.123  24.666  -2.283   0.021   0.451   0.528
E W  2X0BB                268    0.406  97.523  -0.776   0.021   0.451   0.528
E V  2X0BB                269    0.269  41.361  -1.208   0.021   0.279   0.699
E D  2X0BB                270    0.432  62.194  -1.243   0.018   0.047   0.935
E N  2X0BB                271    0.774 113.240  -0.396   0.018   0.019   0.964
E S  2X0BB                272    0.601  70.472  -1.083   0.018   0.019   0.964
E T  2X0BB                273    0.518  71.791  -0.204   0.004   0.197   0.799
E S  2X0BB                274    0.523  61.260   0.056   0.004   0.616   0.381
E V  2X0BB                275    0.272  41.791   0.687   0.002   0.816   0.182
E S  2X0BB                276    0.490  57.393   0.239   0.002   0.816   0.182
B V  2X0BB                277    0.122  18.767   0.577   0.018   0.846   0.136
E P  2X0BB                278    0.356  50.502  -0.261   0.021   0.756   0.223
B M  2X0BB                279    0.193  38.619   0.231   0.018   0.846   0.136
B L  2X0BB                280    0.050   9.100  -0.494   0.001   0.900   0.099
B S  2X0BB                281    0.206  24.120   0.275   0.001   0.959   0.040
B G  2X0BB                282    0.269  21.178  -0.782   0.001   0.900   0.099
E M  2X0BB                283    0.442  88.364  -0.648   0.003   0.718   0.279
B G  2X0BB                284    0.261  20.572  -1.642   0.004   0.616   0.381
E T  2X0BB                285    0.435  60.321  -0.585   0.004   0.616   0.381
B F  2X0BB                286    0.074  14.832  -0.365   0.004   0.616   0.381
E Q  2X0BB                287    0.277  49.436   0.463   0.023   0.655   0.322
B H  2X0BB                288    0.101  18.336   0.142   0.021   0.756   0.223
B W  2X0BB                289    0.152  36.484  -0.545   0.021   0.756   0.223
B S  2X0BB                290    0.255  29.863  -0.645   0.021   0.756   0.223
B D  2X0BB                291    0.100  14.381  -0.645   0.004   0.616   0.381
E I  2X0BB                292    0.582 107.707  -0.620   0.020   0.205   0.775
E Q  2X0BB                293    0.652 116.447  -0.644   0.018   0.088   0.893
E D  2X0BB                294    0.253  36.457   0.029   0.005   0.045   0.951
E N  2X0BB                295    0.528  77.255   0.369   0.004   0.085   0.910
B F  2X0BB                296    0.045   9.132  -0.071   0.004   0.616   0.381
E S  2X0BB                297    0.292  34.199   1.249   0.001   0.900   0.099
B V  2X0BB                298    0.071  10.943   0.069   0.001   0.959   0.040
B T  2X0BB                299    0.049   6.782   0.024   0.001   0.959   0.040
B Q  2X0BB                300    0.216  38.595   0.599   0.000   0.983   0.017
B V  2X0BB                301    0.125  19.136   1.018   0.001   0.959   0.040
E P  2X0BB                302    0.296  42.045  -0.311   0.003   0.718   0.279
B F  2X0BB                303    0.134  26.894  -0.468   0.004   0.514   0.481
E T  2X0BB                304    0.596  82.721   0.222   0.005   0.262   0.733
E E  2X0BB                305    0.735 128.474  -1.979   0.005   0.045   0.951
E S  2X0BB                306    0.595  69.793  -0.725   0.005   0.045   0.951
B A  2X0BB                307    0.233  25.666   0.430   0.004   0.420   0.576
B C  2X0BB                308    0.086  12.018  -0.501   0.001   0.900   0.099
B L  2X0BB                309    0.021   3.863   0.916   0.001   0.959   0.040
B L  2X0BB                310    0.032   5.804   0.569   0.001   0.959   0.040
B L  2X0BB                311    0.017   3.076   1.246   0.000   0.983   0.017
B I  2X0BB                312    0.021   3.867   0.764   0.001   0.900   0.099
B Q  2X0BB                313    0.027   4.840   0.694   0.004   0.616   0.381
B P  2X0BB                314    0.049   6.996  -0.242   0.004   0.138   0.858
E H  2X0BB                315    0.417  75.834   0.538   0.018   0.019   0.964
E Y  2X0BB                316    0.499 106.743  -0.244   0.058   0.017   0.925
E A  2X0BB                317    0.500  55.089  -1.004   0.181   0.016   0.803
E S  2X0BB                318    0.478  56.010  -0.729   0.257   0.016   0.727
E D  2X0BB                319    0.386  55.565   0.166   0.176   0.004   0.820
B L  2X0BB                320    0.060  10.913  -0.092   0.923   0.002   0.076
E D  2X0BB                321    0.545  78.477   1.542   0.923   0.002   0.076
E K  2X0BB                322    0.513 105.442   1.447   0.923   0.002   0.076
B V  2X0BB                323    0.047   7.255  -0.749   0.923   0.002   0.076
B E  2X0BB                324    0.195  34.101  -0.177   0.858   0.002   0.139
E G  2X0BB                325    0.677  53.280   1.011   0.694   0.003   0.303
E L  2X0BB                326    0.439  80.326   0.290   0.406   0.004   0.590
B T  2X0BB                327    0.107  14.883  -0.462   0.176   0.004   0.820
E F  2X0BB                328    0.430  86.241   0.223   0.018   0.019   0.964
E Q  2X0BB                329    0.393  70.261   0.326   0.858   0.002   0.139
E Q  2X0BB                330    0.758 135.432   0.625   0.923   0.002   0.076
E N  2X0BB                331    0.243  35.531   1.185   0.923   0.002   0.076
B S  2X0BB                332    0.029   3.446   0.248   0.970   0.001   0.030
E L  2X0BB                333    0.432  79.136   1.662   0.970   0.001   0.030
E N  2X0BB                334    0.544  79.598   1.682   0.970   0.001   0.030
B W  2X0BB                335    0.075  17.989  -0.206   0.923   0.002   0.076
B M  2X0BB                336    0.176  35.158  -0.083   0.858   0.002   0.139
E K  2X0BB                337    0.660 135.803   0.306   0.782   0.003   0.216
E K  2X0BB                338    0.481  98.962  -0.067   0.502   0.002   0.495
B L  2X0BB                339    0.195  35.686  -0.815   0.257   0.016   0.727
E S  2X0BB                340    0.497  58.190  -0.274   0.052   0.084   0.864
E P  2X0BB                341    0.492  69.758  -0.588   0.020   0.205   0.775
E R  2X0BB                342    0.449 102.707  -0.126   0.004   0.420   0.576
E T  2X0BB                343    0.409  56.756   0.332   0.002   0.816   0.182
B I  2X0BB                344    0.044   8.214  -0.209   0.001   0.900   0.099
E H  2X0BB                345    0.318  57.808   0.260   0.001   0.959   0.040
B L  2X0BB                346    0.041   7.470  -1.083   0.001   0.959   0.040
B T  2X0BB                347    0.177  24.564  -0.099   0.001   0.900   0.099
B M  2X0BB                348    0.084  16.888   0.005   0.004   0.420   0.576
B P  2X0BB                349    0.155  22.009  -0.606   0.004   0.197   0.799
E Q  2X0BB                350    0.235  42.007   0.109   0.003   0.718   0.279
B L  2X0BB                351    0.050   9.173  -0.347   0.001   0.900   0.099
E V  2X0BB                352    0.330  50.721   1.101   0.001   0.900   0.099
B L  2X0BB                353    0.088  16.186  -0.015   0.011   0.918   0.071
E Q  2X0BB                354    0.527  94.086   0.076   0.021   0.756   0.223
E G  2X0BB                355    0.372  29.253  -1.041   0.022   0.552   0.426
E S  2X0BB                356    0.550  64.413  -0.278   0.021   0.451   0.528
E Y  2X0BB                357    0.270  57.656   0.175   0.064   0.216   0.721
E D  2X0BB                358    0.478  68.837   0.787   0.191   0.086   0.723
B L  2X0BB                359    0.052   9.485  -0.439   0.782   0.003   0.216
E Q  2X0BB                360    0.403  71.922   1.385   0.858   0.002   0.139
E D  2X0BB                361    0.519  74.831   0.663   0.879   0.010   0.111
B L  2X0BB                362    0.128  23.400  -0.330   0.879   0.010   0.111
B L  2X0BB                363    0.053   9.778  -0.702   0.879   0.010   0.111
E A  2X0BB                364    0.524  57.778   0.480   0.879   0.010   0.111
E Q  2X0BB                365    0.608 108.625   0.157   0.717   0.014   0.269
B A  2X0BB                366    0.159  17.566   0.292   0.339   0.016   0.645
E E  2X0BB                367    0.502  87.630   0.160   0.058   0.017   0.925
B L  2X0BB                368    0.051   9.411   0.087   0.115   0.016   0.868
E P  2X0BB                369    0.434  61.556   0.822   0.268   0.043   0.689
E A  2X0BB                370    0.524  57.701   0.092   0.502   0.102   0.396
B I  2X0BB                371    0.129  23.865  -0.059   0.455   0.046   0.498
B L  2X0BB                372    0.087  16.003  -0.041   0.386   0.097   0.517
E H  2X0BB                373    0.501  91.059  -0.461   0.184   0.043   0.773
E T  2X0BB                374    0.607  84.191  -1.684   0.113   0.043   0.844
E E  2X0BB                375    0.446  77.881  -0.877   0.053   0.043   0.903
B L  2X0BB                376    0.293  53.685  -1.886   0.058   0.017   0.925
E N  2X0BB                377    0.458  67.007  -1.126   0.053   0.043   0.903
B L  2X0BB                378    0.077  14.099  -1.006   0.053   0.043   0.903
E Q  2X0BB                379    0.468  83.638  -0.861   0.018   0.088   0.893
E K  2X0BB                380    0.400  82.321  -0.892   0.018   0.088   0.893
B L  2X0BB                381    0.103  18.841  -0.860   0.052   0.084   0.864
E S  2X0BB                382    0.277  32.453  -1.233   0.053   0.043   0.903
E N  2X0BB                383    0.672  98.425  -1.002   0.018   0.019   0.964
E D  2X0BB                384    0.580  83.607  -1.792   0.018   0.019   0.964
E R  2X0BB                385    0.529 121.210  -1.130   0.058   0.017   0.925
B I  2X0BB                386    0.247  45.677  -0.942   0.113   0.087   0.800
E R  2X0BB                387    0.261  59.723   0.485   0.148   0.418   0.435
B V  2X0BB                388    0.091  13.971  -0.440   0.169   0.612   0.219
B G  2X0BB                389    0.229  17.999  -0.941   0.159   0.516   0.325
E E  2X0BB                390    0.319  55.799   0.724   0.169   0.612   0.219
B V  2X0BB                391    0.029   4.488   0.099   0.087   0.683   0.230
B L  2X0BB                392    0.135  24.719   0.753   0.018   0.846   0.136
B N  2X0BB                393    0.064   9.355   0.194   0.011   0.918   0.071
B S  2X0BB                394    0.166  19.397   1.279   0.011   0.918   0.071
B I  2X0BB                395    0.022   4.070   0.481   0.001   0.959   0.040
B F  2X0BB                396    0.142  28.439   1.259   0.000   0.983   0.017
B F  2X0BB                397    0.028   5.579   0.720   0.000   0.983   0.017
B E  2X0BB                398    0.220  38.434   0.634   0.001   0.959   0.040
B L  2X0BB                399    0.055  10.052   0.228   0.001   0.900   0.099
E E  2X0BB                400    0.277  48.392   0.792   0.004   0.514   0.481
E A  2X0BB                401    0.387  42.691  -1.166   0.021   0.279   0.699
E D  2X0BB                402    0.598  86.143  -0.120   0.020   0.205   0.775
E E  2X0BB                403    0.423  73.863  -1.413   0.020   0.205   0.775
E R  2X0BB                404    0.404  92.447  -0.239   0.066   0.296   0.638
E E  2X0BB                405    0.581 101.501  -0.263   0.066   0.296   0.638
E P  2X0BB                406    0.336  47.650  -0.739   0.064   0.216   0.721
E T  2X0BB                407    0.397  55.050  -1.175   0.064   0.216   0.721
E E  2X0BB                408    0.495  86.424  -1.092   0.064   0.216   0.721
E S  2X0BB                409    0.348  40.774  -0.486   0.021   0.279   0.699
B T  2X0BB                410    0.250  34.647  -1.409   0.022   0.359   0.619
E Q  2X0BB                411    0.371  66.332  -0.882   0.022   0.552   0.426
B Q  2X0BB                412    0.294  52.437  -1.515   0.023   0.655   0.322
E L  2X0BB                413    0.379  69.450  -1.465   0.022   0.552   0.426
E N  2X0BB                414    0.482  70.638  -2.024   0.021   0.451   0.528
E K  2X0BB                415    0.421  86.600  -1.303   0.020   0.205   0.775
E P  2X0BB                416    0.520  73.802  -1.778   0.004   0.085   0.910
E E  2X0BB                417    0.592 103.440  -0.824   0.004   0.085   0.910
E V  2X0BB                418    0.294  45.126  -0.566   0.004   0.138   0.858
B L  2X0BB                419    0.264  48.393  -0.162   0.004   0.616   0.381
E E  2X0BB                420    0.393  68.570   1.540   0.001   0.900   0.099
B V  2X0BB                421    0.034   5.210   0.090   0.001   0.959   0.040
E T  2X0BB                422    0.261  36.270   1.087   0.001   0.959   0.040
B L  2X0BB                423    0.025   4.559   0.742   0.002   0.816   0.182
B N  2X0BB                424    0.251  36.790   0.056   0.005   0.262   0.733
B R  2X0BB                425    0.185  42.319   1.219   0.005   0.045   0.951
B P  2X0BB                426    0.077  10.926   0.139   0.004   0.138   0.858
B F  2X0BB                427    0.026   5.258   0.977   0.002   0.816   0.182
B L  2X0BB                428    0.033   6.134   0.555   0.001   0.959   0.040
B F  2X0BB                429    0.027   5.339   0.961   0.001   0.959   0.040
B A  2X0BB                430    0.020   2.237   1.451   0.001   0.959   0.040
B V  2X0BB                431    0.019   2.843   1.340   0.001   0.959   0.040
B Y  2X0BB                432    0.120  25.601   0.858   0.000   0.983   0.017
B D  2X0BB                433    0.142  20.477   0.545   0.001   0.900   0.099
E Q  2X0BB                434    0.443  79.084   0.922   0.004   0.138   0.858
E S  2X0BB                435    0.650  76.180  -0.201   0.005   0.045   0.951
E A  2X0BB                436    0.280  30.878  -0.498   0.005   0.045   0.951
E T  2X0BB                437    0.413  57.311   0.612   0.005   0.045   0.951
B A  2X0BB                438    0.092  10.094   1.248   0.004   0.420   0.576
B L  2X0BB                439    0.025   4.651   1.008   0.002   0.816   0.182
B H  2X0BB                440    0.029   5.202   1.034   0.001   0.959   0.040
B F  2X0BB                441    0.034   6.764  -0.175   0.001   0.959   0.040
B L  2X0BB                442    0.038   6.958   0.310   0.001   0.959   0.040
B G  2X0BB                443    0.025   1.936  -0.027   0.001   0.959   0.040
B R  2X0BB                444    0.153  34.945   1.141   0.001   0.959   0.040
B V  2X0BB                445    0.046   7.086   0.328   0.001   0.900   0.099
B A  2X0BB                446    0.209  23.043   0.480   0.003   0.718   0.279
B N  2X0BB                447    0.246  35.970   0.616   0.005   0.262   0.733
B P  2X0BB                448    0.172  24.336  -0.554   0.018   0.088   0.893
E L  2X0BB                449    0.495  90.689  -0.209   0.018   0.047   0.935
E S  2X0BB                450    0.656  76.848  -0.237   0.018   0.047   0.935
E T  2X0BB                451    0.604  83.789  -2.101   0.018   0.019   0.964
E A  2X0BB                452    0.781  86.099  -1.897   0.003   0.003   0.994

Explain the output.  Go back.
```

---
